# MicroRNA and Cancer: Tiny Molecules with Major Implications

**DOI:** 10.2174/138920208784139555

**Published:** 2008-04

**Authors:** Timothy G VandenBoom II, Yiwei Li, Philip A Philip, Fazlul H Sarkar

**Affiliations:** 1 Department of Pathology, Wayne State University School of Medicine, Detroit, MI, USA; 2 Division of Hematology and Oncology, Internal Medicine, Karmanos Cancer Institute, Wayne State University School of Medicine, Detroit, MI, USA

**Keywords:** microRNA, biogenesis, post-transcriptional regulation, cancer, diagnosis, prognosis, therapy.

## Abstract

Cancer is currently a major public health problem and, as such, emerging research is making significant progress in identifying major players in its biology. One recent topic of interest involves microRNAs (miRNAs) which are small, non-coding RNA molecules that inhibit gene expression post-transcriptionally. They accomplish this by binding to the 3’ untranslated region (3’UTR) of target messengerRNA (mRNA), resulting in either their degradation or inhibition of translation, depending on the degree of complementary base pairing. They are transcribed by RNA polymerase II and are formed into mature miRNAs *via *two steps, each catalyzed by a different ribonuclease III (RNaseIII). Cross-species comparisons demonstrate that miRNAs are evolutionarily conserved and play important roles in a wide array of normal biological processes. Importantly, aberrant miRNA expression is correlated with human disease, especially in the development of cancer. Recent research has identified targets and functions of miRNAs, illustrating that some are oncogenic in nature while others show tumor suppressor activity. The miRNAs have also been characterized as having high potential in the clinical arena and, as such, have been a target for exploitation toward cancer therapy. Not only has it been shown that miRNA expression profiles may prove useful as diagnostic and prognostic markers in cancer, various miRNA-based therapies show promise as well. It is anticipated that further research will elucidate the benefits of using miRNAs as clinical agents in the battle against cancer and other chronic diseases.

## INTRODUCTION

Cancer is currently one of the leading causes of death in the United States and other developed countries, and thus has caused many public health concerns in the present and will continue as a major health concern in the future unless significant breakthroughs are made in the therapeutic arenas for the treatment of not only cancers, but other chronic diseases that are intimately involved with the etiology of cancer. Projected cancer statistics for 2007 developed by the American Cancer Society clearly illustrate the effect of cancer on the population. It was estimated that in the United States, about 1.44 million new cases of cancer would arise in 2007. Also, the American Cancer Society predicted there would be about 559,650 cancer-related deaths in 2007, which corresponds to over 1,500 deaths per day [1]. These statistics, among many others, display the degree of this alarming problem and suggest that more diagnostic, prognostic, and therapeutic interventions need to be developed in order to sufficiently offset the growing number of cancer-related deaths for the betterment of public health.

Normal cells in the human body incorporate several mechanisms that ensure proper cell division, differentiation, and apoptosis. Various regulatory factors control the expression of genes such as tumor suppressor genes and oncogenes, allowing for the timely and coordinated execution of these processes. In cancer, however, these genes do not function properly, causing these processes to become uncontrolled, resulting in tumor formation [2]. Recent research has led to significant progress in terms of elucidating the molecular mechanisms and damaged genes involved in cancer. One such example is the discovery of microRNAs (miRNAs), leading to a sudden escalation in research implicating these tiny RNA molecules as integral players in cancer biology.

miRNAs were first discovered in 1993 while studying *Caenorhabditis elegans*. The first miRNA, lin-4, was characterized as a small, non-coding RNA molecule that played a role in development by negatively regulating lin-14 expression [3, 4]. The second miRNA, let-7, was discovered in 2000 in *C. elegans* [5]. The discovery of these two miRNAs stimulated a huge explosion in miRNA research. In the past six years, over 4500 miRNAs have been identified in vertebrates, flies, worms, plants and viruses [6-8]. This number is likely to grow in the future because further innovative research is continuing at a very rapid pace. 

## miRNA BIOGENESIS

**Fig. (1):**
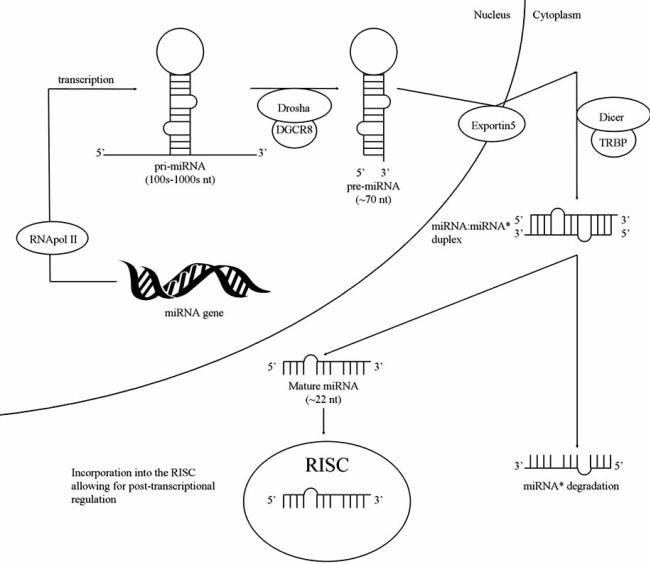
**miRNA Biogenesis.** RNA polymerase II transcribes the miRNA gene and forms a pri-miRNA transcript, ranging anywhere from hundreds to thousands of nucleotides in length. This is processed by the RNase III Drosha and DGCR8, yielding the ~70 nucleotide premiRNA. The pre-miRNA is transported to the cytoplasm *via* Exportin 5 where it is processed by the RNase III Dicer and TRBP, releasing a miRNA:miRNA* duplex, about 22 nucleotides in length. One strand of this duplex is degraded while the other strand, the mature miRNA, preferentially enters the RNA-induced silencing complex (RISC), allowing it to engage in post-transcriptional regulation of functional mRNAs.

miRNA biogenesis (Fig. **1**) begins with transcription by RNA polymerase II, yielding a primary miRNA (pri-miRNA) which ranges anywhere from hundreds to thousands of nucleotides in length [9]. Two steps, each accomplished by a different ribonuclease III (RNaseIII), are needed in order to yield a functional, mature miRNA. The two steps described below focus on humans. The first step is catalyzed by the nuclear RNaseIII, Drosha, which cleaves the pri-miRNA to yield the precursor miRNA (pre-miRNA), about 70 nucleotides in length. Drosha requires the help of the dsRBD protein, DGCR8, in order to ensure accurate and efficient processing of the pri-miRNA into the pre-miRNA [10-15]. The nuclear export factor Exportin 5 binds the pre-miRNA and transports it to the cytoplasm, where the second step in miRNA biogenesis takes place [16-18]. The second RNaseIII, Dicer, processes the pre-miRNA and yields an RNA duplex about 22 nucleotides in length. This enzyme needs the help of another dsRBD protein, the *trans-activator RNA (tar)-*binding protein (TRBP) [19-23]. One strand of this duplex is the mature miRNA which preferentially enters the RNA-induced silencing complex (RISC) [24-26], enabling it to engage in its main duty, post-transcriptional regulation of functional mRNA.

## MODES OF miRNA ACTION

**Fig. (2):**
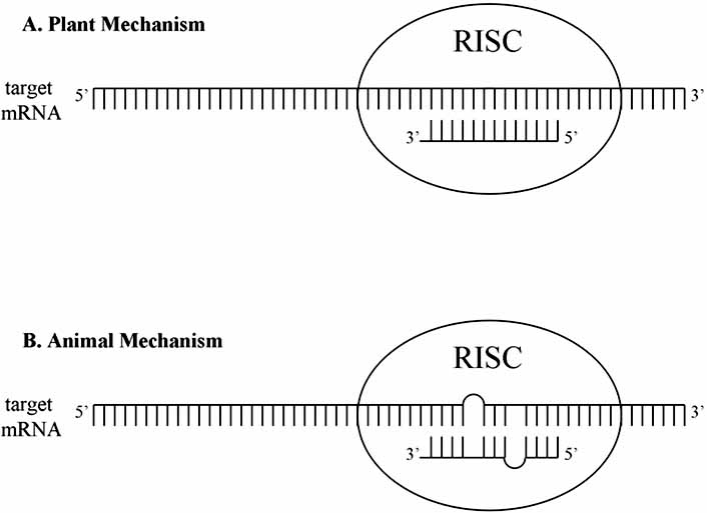
**Modes of miRNA Action.** *A*: complimentary base pairing of miRNA with mRNA, which results in the degradation of mRNAs, a common mechanism in plants. *B*: imperfect base pairing of miRNA with mRNA results in translational inhibition, the typical mode of action in animals.

The miRNAs play a role in post-transcriptional regulation by binding to the 3’ untranslated region (3’UTR) of target messengerRNA (mRNA). Either perfect or near perfect complimentary base pairing results in the degradation of the mRNA, while partial base pairing leads to translational inhibition and silencing of the targeted message [27-29]. It has been observed that plant miRNAs engage in the first mechanism while animal miRNAs primarily employ the latter mechanism, tolerant of mismatches (Fig. **2**). The imperfect miRNA-mRNA complementarity in human cells is usually composed of matched nucleotides in the 5’ portion of the miRNA, termed the seed sequence (positions 2-7), with mismatches at positions 10 and 11 [30-32]. In humans, this complex leads to the inhibition of target gene protein translation and only very rarely causes degradation of the mRNA [33]. Although this imperfect binding of human miRNAs is commonly accepted, there is still much debate regarding how the mi-RISC-mRNA complex actually triggers the silencing of mRNA gene expression. The various propositions of this mechanism have recently been put forward as documented in recent review articles [34, 35].

## ROLES of miRNA IN NORMAL PHYSIOLOGY AND PATHOLOGY

The miRNAs have been discovered and studied in a wide range of species which has allowed for cross-species comparisons yielding very important findings. These comparisons have shown that many miRNAs are conserved across animal species, indicating that they are fundamental aspects of normal biological processes. In fact, predictions of miRNA targets in various computer modeling studies have suggested that up to 30% of human protein coding genes may be regulated by miRNAs [33], making them one of the largest classes of regulatory molecules in humans. miRNAs have been implicated in a wide array of cell functions in both vertebrates and invertebrates, including processes integral to normal physiology such as cell proliferation, differentiation, death, stress resistance, and fat metabolism [6]. With respect to vertebrates, genetic knockouts in animals have been useful models to study miRNA function. For example, genetic removal of the RNaseIII Dicer in zebrafish and mice has shown the vital roles of miRNAs that are essential in many cellular processes such as in the development of a species. An animal deficient in this important enzyme is unable to produce new miRNAs. The loss of Dicer caused lethality in both species, indicating that Dicer plays a vital modulatory role during development [36-39]. Other similar studies have displayed important roles of miRNAs in processes of stem cell differentiation, cardiac and skeletal muscle development, neurogenesis, hematopoiesis, and immune function [40-44]. Along with many normal processes, miRNAs or the processing of miRNAs has been shown to be key players in several human diseases. Among the many, some include Spinal muscular atrophy, fragile X mental retardation, Parkinson’s disease, and DiGeorge syndrome [13, 45-47]. Recent research has also identified the critical involvement of miRNAs in cancer, the main focus of this article.

## IMPLICATIONS of miRNAS IN CANCER

Defects in normal cell processes such as differentiation, proliferation, and apoptosis are all well-known to be involved in cancer pathogenesis. One reason the connection between miRNA and cancer was initially made is because miRNAs were found to be involved in many of these processes. This connection initiated research which further reinforced the correlation between miRNAs and cancer development. Researchers discovered that there is aberrant miRNA expression when comparing various types of cancer with normal tissues. Although the association between miRNA and cancer was initially suggested, the question still remained regarding whether the altered miRNA expression was a cause or a consequence of cancer [33]. Also, not much was known about the specific targets and functions of miRNAs. The first direct evidence for the key role of miRNAs in cancer came from a study in human chronic lymphocytic leukemia (CLL). In this study, Calin *et al*. decided to focus on the deletion at locus 13q14, the most frequent chromosomal abnormality in CLL. Although many studies had focused on this deletion, they all failed to notice consistent involvement of any of the genes located in the deletion locus. They further analyzed this region and found that genes for two miRNAs, miR-15 and miR-16, were located exactly in the deleted region. Additional investigation of these two miRNAs revealed that they both showed a significant reduction in expression when compared to their normal tissue counterparts [48]. Although future research is needed to determine their mechanism of action and their exact role in CLL, these findings suggest that these two miRNAs play a causal role in CLL. This study sparked significant miRNA research, leading to the discovery of exact targets and functions of miRNAs in relation to cancer as well as clinical significance involving the diagnostic, prognostic, and therapeutic value of miRNAs. 

Some miRNAs are thought to have oncogenic activity while others have tumor suppressor activity (Table **1**). It is important to note that these distinctions may not be so strict and that some miRNAs may express either activity, depending on the situation and tissue type. Nevertheless, the majority of recent research provides results that point toward one category or the other. It is also possible to group miRNAs based on their various functions (Table **2**). Some play a single role while others contribute to cancer through multiple cellular functions. Although there has been significant progress in regards to many miRNAs, only the most highly studied and well-described miRNAs in each category will be discussed below. 

## miRNAS WITH ONCOGENIC ACTIVITY

### miR-17-92 Cluster

Oncogenic miRNAs are up-regulated in cancer and contribute to its pathology through various mechanisms such as targeting tumor suppressor genes. Falling into this category is the group of six miRNAs termed the miR-17-92 cluster which is made up of miR-17, miR-18a, miR-19a, miR-20a, miR-19b-1, and miR-92-1 [49]. This cluster is encoded by the *c13orf25 *gene located at 13q31, a genomic locus that is frequently amplified in cases of B-cell lymphoma and other various tumor types. He *et al*. researched this further by comparing B-cell lymphoma samples and cell lines to normal B-cells [50]. The comparison displayed a high level of expression of the six mature miRNAs in the cancer cell lines, prompting the hypothesis that the miR-17-92 cluster may contribute to tumor development. These researchers tested this directly in a mouse model of B-cell lymphoma with some of the transgenic animals carrying the *c-myc* oncogene. After inducing expression of the miR-17-92 cluster, they noticed that animals expressing both *c-myc* and the miRNA cluster developed highly malignant, disseminated lymphomas capable of evading apoptosis [50]. Similarly, Dews *et al*. investigated the role of the miR-17-92 cluster in Myc-induced tumorigenesis [51]. It is known that Myc enhances angiogenesis by down regulating antiangiogenic thrombospondin-1 (Tsp1) and related proteins, such as connective tissue growth factor (CTGF). They sought to determine whether Myc-induced up-regulation of the miR-17-92 cluster is directly responsible for the down regulation of these anti-angiogenic factors. Anti-sense 2’-*O*-methyl oligoribonucleotides targeting the miRNAs from this cluster caused partial restoration of Tsp1 and CTGF expression. They also generated cells exhibiting over-expression of this cluster through transduction and found lower levels of the two proteins, especially for CTGF [51], suggesting that the effects of the miR-17-92 cluster are angiogenic and oncogenic.

### miR-155

Another miRNA that seems to fit into the oncogenic group is miR-155. The *BIC* gene, elevated in cancer, has been shown to produce non-protein coding RNA. Recently, in mice, miR-155 was found to be encoded within the phylogenetically conserved region of *BIC* RNA [52]. Therefore, Eis *et al*. hypothesized that miR-155 could be responsible for the oncogenic activity of *BIC *RNA and decided to investigate it further. In clinical B-cell lymphomas they found that *BIC *RNA, and to an even greater extent miR-155, both had increased accumulation when compared to controls [53]. Furthermore, many studies using miRNA expression profiling have recognized the altered expression of miR-155 in a variety of cancers when compared to their normal counterparts. For example, a study by Iorio *et al*. [54] analyzed 76 breast cancer and 10 normal breast samples and compared their miRNA expression profiles in order to identify which miRNAs were significantly deregulated in breast cancer. The miR-155 was found to be ranked as one of the top five differentially expressed miRNAs. It was consistently up-regulated, suggesting a potential oncogenic role in cancer [54]. Other studies have also recognized the up-regulation of miR-155 in cancers [55-58]. 

Another study identified one of the specific targets of miR-155. Tumor protein 53-induced nuclear protein 1 (TP53INP1) is a pro-apoptotic stress-induced p53 target gene. A study by Gironella *et al*. [59] showed that TP53INP1 expression is significantly reduced in pancreatic ductal adenocarcinoma (PDAC) and therefore, they decided to elucidate the molecular mechanism by which the expression is deregulated. Since they found that only protein levels are changed and not mRNA levels, they hypothesized that a post-transcriptional mechanism is responsible for regulating expression and that a miRNA may function as a key player in the regulation of TP53INP1. Bioinformatic approaches revealed miR-155 as a probable target. Therefore, in order to test this, they transfected Capan2 cells with an anti-miR-155 oligonucleotide which caused re-expression of TP53INP1 and a significant increase in apoptosis, demonstrating that TP53INP1 is a target of miR-155 [59]. 

### miR-21

An additional well-studied miRNA with oncogenic activity is miR-21. Similar to miR-155, this miRNA is deregulated in a variety of cancers. In the same study by Iorio *et al*. as mentioned above, miR-21 also ranked as one of the top five differentially expressed miRNAs and was consistently up-regulated, suggesting an oncogenic role [54]. In a study by Chan *et al*., miRNA expression analysis of human glioblastoma tumor tissues, early-passage glioblastoma cultures, and six established glioblastoma cell lines displayed strikingly elevated miR-21 levels when compared to controls [60]. To study the biological significance of this elevation, they utilized a loss-of-function approach in which they employed two different anti-sense oligonucleotide strategies, both of which produced similar results. In both cases, inhibition of miR-21 caused an increase in apoptosis due to activation of caspases, indicating the oncogenic role of this miRNA in cancer [60]. Si *et al*. found increased expression of miR-21 to be present in breast tumor tissues as well [61]. To test whether miR-21 may function as an oncogene, they used a knockdown approach using an anti-miR-21 inhibitor. The results showed inhibition of cell growth *in vitro *as well as inhibition of tumor growth *in vivo* in a xenograft carcinoma mouse model after knockdown of miR-21. Finally, the use of anti-miR-21 displayed increased cell apoptosis, which was associated with down regulation of bcl-2 expression [61]. Further studies sought to elucidate exact targets of miR-21 as presented below. Pdcd4, a novel tumor suppressor, is down regulated in various types of cancer, especially lung and colorectal, which is associated with poor patient prognosis. Since little was known about the regulatory mechanisms of Pdcd4 in cancer, Asangani *et al*. sought to determine the role, if any, of miRNAs in the regulation of this tumor suppressor as well as their role in invasion and intravasation [62]. They screened the 3’UTR of Pdcd4 in search of any complementarity to the seed sequences of known miRNAs and found an exact match target sequence for miR-21. It was also found that Pdcd4 is inversely correlated with miR-21 levels in colorectal cancer cell lines as well as the resected tumor tissues of 22 colorectal cancer patients. Moreover, anti-miR-21 transfected cells exhibited increased Pdcd4 expression and reduced invasion and intravasation, while over-expression of miR-21 in these cells showed the opposite effect [62], suggesting that Pdcd4 is a target of miR-21.

## miRNAS WITH TUMOR SUPPRESSOR ACTIVITY

### miR-15 and miR-16

In contrast to the oncogenic miRNAs, other miRNAs are considered to have tumor suppressor activity and are down regulated in cancer. Along with the initial findings discussed earlier, research has further implicated miR-15 and miR-16 as miRNAs with tumor suppressor activity. One study by Cimmino *et al*. investigated the role of these miRNAs in relation to bcl-2, an anti-apoptotic player in survival pathways which has been found to be over-expressed in a variety of human cancers [63]. They found that the levels of miR-15 and miR-16 are inversely correlated with bcl-2 expression in CLL cells. Also, they determined the effects of transfection of these miRNAs on bcl-2 expression. In a cell line with high bcl-2 and no expression of these miRNAs, they found that transfection of miR-15 and miR-16 caused a significant reduction in bcl-2 expression. Finally, they identified the biological effects of down regulating bcl-2 by miR-15 and miR-16. They found that bcl-2 down regulation by these two miRNAs caused apoptosis in a CLL cell line [64].

### let-7 Family

One of the most studied miRNA groups with tumor suppressor potential is the let-7 family. The let-7 family consists of let-7, miR-48, miR-84, and miR-241 [49]. Early research findings showed reduced let-7 expression in cancer and further studies suggested the role and biological significance of let-7 in prognosis. Takamizawa *et al*. analyzed 20 human lung cancer cell lines as well as 16 primary human lung cancer tissues and noticed that the reduction in let-7 expression was a frequent occurrence when compared to controls [65]. Furthermore, they collected 143 lung cancer specimens from patients that had undergone curative resection and analyzed them using real-time reverse transcription-PCR. Unsupervised hierarchical analysis classified the specimens into two groups, displaying significantly shorter post-operative survival in cases with reduced let-7 expression. Biological significance of this reduction was also achieved by over-expressing let-7f in the A549 lung adenocarcinoma cell line which resulted in a 78.6% reduction in the number of colonies [65]. Similar to the other highly studied miRNAs, many miRNA expression studies have revealed reduced expression of the let-7 family in various types of cancer. Research has also determined exact targets of this tumor suppressing let-7 family. Johnson *et al*. identified the regulation of *let-60*, the ortholog of the human *RAS* oncogene, by let-7 in *C. elegans *[66]. A computational screen indicated *let-60* as one of the top scoring genes with let-7 family complementary sites in their 3’UTR. Consequently, they decided to extend these results to humans and have identified a potential relationship. They found that all three human *RAS* 3’UTRs contain multiple let-7 family complementary sites, suggesting a possible regulatory role of these miRNAs. Additionally, transfection of HepG2 cells with a let-7a precursor resulted in about 70% reduction of *RAS*. In contrast, transfection of HeLa cells with anti-sense let-7 molecules reduced the expression of let-7 and in turn, allowed for an approximately 70% increase in *RAS* levels [66]. These results suggest a possible mechanism for let-7 in cancer development. Similar elucidation of let-7 targets have occurred in other studies, like the regulation of high-mobility group protein HMGA2 by let-7 [67].

### Roles in the p53 Tumor Suppressor Network

Lastly, of important note is the role of miRNAs in the p53 tumor suppressor network. p53 is a tumor suppressor protein that coordinates cellular responses to various cancer initiating stressors [68, 69]. Since miRNAs often display aberrant expression in cancer, studies hypothesized that miRNAs may be targets of the p53 network in response to stress. In a study by Chang *et al*., the researchers treated cells with the DNA-damaging agent adriamycin and subsequently performed a custom microarray analysis [70]. Among the miRNAs up-regulated upon DNA damage, miR-34a had the largest magnitude of change. They then characterized the structure of the miR-34a primary transcript and promoter and found that it is directly transactivated by p53. Additionally, they found that increased expression of miR-34a resulted in increased apoptosis and also caused changes in the expression of genes related to many cellular processes such as cell-cycle progression, apoptosis, DNA repair, and angiogenesis [70]. Hence, it was concluded that miR-34a plays a very important role in controlling gene expression of the p53 tumor suppressor network components. Another study shows similar results [71] and others highlighted the importance of miRNAs like let-7 and miR-15/16 as important players in the p53 tumor suppressor network [72].

## miRNA AND THE CLINICAL CANCER

### Diagnostic Value

Now the question arises of how and to what extent miRNAs will be utilized and translated into the clinical setting. Recent research indicates that miRNAs will most likely have diagnostic, prognostic, and therapeutic value. Many studies have displayed that miRNA expression profiles can be used to differentiate different types of tissue. For example, one study by Lee *et al*. completed real-time PCR profiling of over 200 miRNA precursors in specimens of human pancreatic adenocarcinoma, paired benign tissue, normal pancreatic tissue, pancreatitis, and a variety of pancreatic cell lines. Hierarchical clustering was able to differentiate the tumor tissue from normal pancreatic tissue, pancreatitis, and cell lines. Also, using the prediction analysis of microarrays (PAM) algorithm, researchers determined whether or not miRNA expression data could successfully predict which class the samples fit in. PAM correctly classified all of the tested tumor samples and 11 of 15 of the tested benign samples [73]. Another study reported by Porkka *et al*. involved expression profiling of 319 miRNAs in 6 prostate cancer cell lines, 9 prostate cancer xenograft samples, and 13 clinical prostate tissue samples (4 benign prostatic hyperplasia, 5 untreated prostate carcinomas, and 4 hormone-refractory prostate carcinomas). This was done in order to determine the prostate cancer miRNA expression profile and to use this information in classifying prostate tumors. They found differential expression of 51 miRNAs between benign tumors and carcinoma tumors. Also, hierarchical clustering of the tumor samples successfully separated the carcinomas from the benign prostatic hyperplasia samples as well as further separated the carcinoma tumors according to their androgen dependence [74]. The studies described above provide evidence for the potential use of miRNA expression profiling as a novel tool in cancer diagnosis.

### Prognostic Value

The miRNAs may also have significant value in terms of cancer prognosis. Various studies have been successful in correlating miRNA expression levels with survival time. Roldo *et al*. used a custom microarray to determine the miRNA expression in 12 non-tumor pancreas samples and 44 pancreatic primary tumors [75]. Not only did this data reveal the diagnostic value of miRNA expression patterns, they were also able to achieve prognostic value. This group found that the up-regulation of miR-21 is strongly associated with both a high Ki67 proliferation index and presence of liver metastasis [75]. A study by Bloomston *et al*. also demonstrates the prognostic value of miRNAs [76]. This group obtained tissue samples from 65 patients who had undergone resection for PDAC as well as from 42 patients with chronic pancreatitis and tested the expression of miR-196a-2. The results displayed that miR-196a-2 significantly predicted duration of survival. High expression of this miRNA had a median survival of 14.3 months compared with a median of 26.5 months for those with low expression [76]. These two studies, among many others, signify that miRNA expression profiling not only can be used in diagnosis, but can also be used as prognostic markers in cancer. 

### Therapeutic Value

Utilization of miRNAs in therapy is an interesting idea and a major focus of current research activities. One potential strategy is inactivating oncogenic miRNAs. Various studies have employed 2’*-O-*methyl oligonucleotides [77, 78] or locked nucleic acid-modified oligonucleotides [79] to block miRNA function. One such study by Meister *et al*. synthesized anti-miR-21 2’*-O-*methyl oligonucleotides and added them to HeLa extracts. It was found that using this anti-sense oligonucleotide significantly decreased the activity of miR-21 when compared to control oligonucleotides [78]. A similar study completed in mice suggests the potential of this strategy *in vivo *as well [80]. 

Another possible route to follow in terms of therapy is in the areas of restoring down regulated miRNAs that function as tumor suppressors. Johnson *et al*. focused on this concept in his study on elucidating the function of let-7 in human cell proliferation pathways [81]. As stated earlier, let-7 functions as a tumor suppressor and is poorly expressed or deleted in human tumors. In this study they over-expressed let-7 by using exogenously transfected pre-let-7 RNAs and determined the effect on lung and liver cancer cells. They found that over-expressing let-7 consistently reduced the number of proliferating cells in both cell lines [81]. This finding clearly suggests the possible effect of restoring tumor suppressor miRNAs in cancer therapy. 

A third possible strategy is creating synthetic miRNAs to target mRNAs of genes known to contribute to cancer. A study by Tsuda *et al*. focused on the *glioma-associated antigen-1* (*Gli-1*) which plays a role in the sonic hedgehog signaling cascade, a pathway implicated in the growth of a number of human malignancies [82]. Their objective was to determine whether or not synthetic miRNA targeting *Gli-1* mRNA could be used to inhibit tumor cell proliferation in ovarian SK-OV-3 and pancreatic MiaPaCa-2. They found that their designed synthetic miRNAs reduced expression of the Gli-1 protein and inhibited cell proliferation and division. They were able to induce a significant reduction in the number of tumor cells in two cell lines that express high levels of the Gli-1 protein [82]. 

Finally, a fourth possible strategy that has been proposed is utilizing miRNAs as agents to alter resistance to cytotoxic anti-cancer therapy. It has been known that tumor cells use pre-existing survival pathways to evade the cytotoxic effects of anti-cancer agents. A study by Weidhaas *et al*. sought to determine whether miRNAs play a role in this genetic response [83]. They used the lung cancer cell line A549 and the normal lung epithelial cell line CLR2741 and compared the miRNA expression before and after irradiation using a microarray profile. It was found that each member of the let-7 family, except for let-7g, decreased significantly by 2 hours after irradiation in both cell lines. The let-7 family represented a large proportion of the altered miRNAs in response to the radiation. The similarity in response between the cancer cells and normal epithelial cells suggests a highly conserved global miRNA response. This also suggests that miRNAs are possibly integral aspects of the response to cytotoxic insult. Since the let-7 family result was so significant, they decided to determine the effect of altering its expression on the radiation response and cell survival. They were able to create a radiosensitive state *in vitro* in lung cancer cells and *in vivo *in a *C. elegans *model of radiation-induced cell death when they over-expressed the let-7 family of miRNAs. In contrast, decreasing the levels of the let-7 family caused radio-resistance [83]. This study and the others above all provide evidence for the potential use of miRNAs in various therapeutic strategies. 

## CURRENT RESEARCH

Our laboratory has completed some preliminary experiments that resulted in exciting data which reinforces many of the ideas discussed above and further suggest that cutting-edge research in this area is urgently needed. First, we decided to compare a normal pancreatic epithelial cell line (HPDE) with a pancreatic cancer cell line (Panc-1) in order to display the differential miRNA expression between the two. Total RNA was isolated from both cell lines using the *mir*Vana™ miRNA Isolation Kit (Ambion Inc, TX). Upon successful isolation, total RNA samples were sent to LCSciences (Houston, TX) where they completed a dual sample miRNA microarray analysis. Results showed that 121 miRNA transcripts were differentially expressed between the two cell lines (*p* < 0.01). Of these, 75 were up-regulated and 46 were down regulated in Panc-1 when compared to HPDE (Table **3**). Some results did not perfectly match the existing literature, which could be partly due to the lack of comparing multiple cell lines and tissue samples. For example, our results show lower expression of miR-155 in Panc-1 when compared to HPDE, even though most of the literature suggests this miRNA as being up-regulated in cancer and thus known as an oncogenic miRNA. In regards to the miRNAs that were shown to have either oncogenic or tumor suppressor activity in many experimental systems as shown in Table **1**, our results also showed similar up-regulation of the oncogenic miRNAs miR-10a and miR-146b as well as down regulation of the tumor suppressor miRNAs let-7a and let-7f in cancer when compared to the normal cells. Our results were further analyzed by the Bioinformatics Core Facility at Wayne State University in order to determine potential miRNA targets. For example, it was found that the oncogenic miR-10a may target and negatively regulate tyrosine kinase, non-receptor 1 (TNK1). This gene plays a role in the indirect suppression of Ras activity resulting in the inhibition of cell growth. Thus, negative regulation of this gene by miR-10a may counteract its effect and promote Ras activity and cell growth, supporting the suggested oncogenic role of this miRNA in cancer. These results suggest that further in-depth investigations are needed using multiple cancer cell lines and tumors compared to normal cells and normal pancreas in order to firmly establish the role of specific miRNAs toward development of therapeutic strategies in the future. 

Since these preliminary results showed aberrant miRNA expression when comparing the pancreatic cancer cell line to a normal pancreatic epithelial cell line, we decided to perform another study for assessing the effect of two chemopreventive agents on miRNA expression. The pancreatic cancer cell lines Panc-1 and Colo-357 were treated with 25µM B-DIM (3,3’-diindilylmethane from BioResponse, Boulder, CO) or 25µM G2535 (isoflavone mixture from Organic Technologies, Coshocton, OH). After the 72 hour treatment period, cells were subjected to total RNA isolation using the *mir*Vana™ miRNA Isolation Kit (Ambion Inc, TX). Total RNA samples were once again sent to LCSciences (Houston, TX) where they completed miRNA microarrays for each sample. Only taking into account statistically significant data with high signals (signal > 500), the results showed that B-DIM up-regulated 12 miRNA and down regulated 20 miRNA in Panc-1 cells. G2535 increased expression of 19 miRNA and inhibited expression of 28 miRNA in the same cell line (Table **4**). In regards to Colo-357 cells, B-DIM up-regulated 13 miRNA and down regulated 13 miRNA. G2535 increased expression of 34 miRNA and inhibited expression of 22 miRNA in the same cell line (Table **5**). 

The treatments caused changes in miRNA expression that were once again mostly consistent with the known miRNA activities in Table **1**. Although a couple of the miRNAs in our results were not consistent with the literature, most of our data was in direct agreement. For example, we found an increased expression of the oncogenic miR-21 in B-DIM treated Colo-357 which was unexpected, whereas most of the results displayed decreased expression of oncogenic miRNAs and increased expression of tumor suppressor miRNAs upon treatment with either agent, clearly supporting the role of B-DIM or G2535 as cancer preventing agents. It is possible that the up-regulation of miR-21 is due to a pre-existing survival stress response. A similar explanation was previously documented by Rossi *et al*. to explain the increase in miR-21 expression in human colon cancer cells after exposure to 5-fluorouracil *in vitro* [84]. miR-21 has been shown to have proliferative, metastatic, and anti-apoptotic functions in cancer by targeting genes such as PTEN, TPM1, Pdcd4, and maspin. The drug-induced up-regulation of this miRNA could have been due to an intrinsic cellular response aimed at utilizing its functions to evade the cytotoxic effects of this anti-cancer agent. Building on this concept, it is also possible that down regulating this miRNA would chemosensitize the cells and make them more susceptible to the anti-cancer effects of these drugs. Thus, further research is needed to investigate the role of miR-21 and other miRNAs in survival pathways and to discern the potential for exploiting these pathways for the development of a targeted approach for chemosensitization.

Upon treatment of Panc-1 with B-DIM, the oncogenic miRNAs miR-17, miR-20a, miR-106a, miR-221, and miR-222 all showed decreased expression. Treatment of Panc-1 with G2535 elicited decreased expression of the oncogenic miRNAs miR-17, miR-20a, miR-106a, miR-222 and increased expression of the tumor suppressor miRNAs let-7a, let-7d, let-7e and let-7f. In regards to Colo-357, treatment with B-DIM showed decreased expression of the oncogenic miRNAs miR-17, miR-19b-1, miR-20a and miR-106a. Upon treatment of Colo-357 with G2535, the oncogenic miRNAs miR-17, miR-19b-1, miR-20a, miR-106a, miR-200b and miR-221 showed decreased expression and the tumor suppressor miRNAs let-7a, let-7b, let-7c, let-7d, let-7f, let-7i and miR-16-1 showed increased expression. Once again, it is important to note that our results are consistent especially when compared to the results reported in Table **1**. Other important miRNAs were also up-regulated and down regulated after treatment that are not mentioned in Table **1**, suggesting that further in-depth research in this area is urgently needed, which is likely to assist in pursuing newer avenues for the treatment of pancreatic and other cancers using non-toxic natural products alone or in combination with conventional therapeutics. 

It was also interesting when we examined the differences between B-DIM treatment and G2535 treatment. In both cell lines, treatment with G2535 caused a larger number of miRNAs that were significantly changed when compared to B-DIM treatment. It is also noteworthy that B-DIM treatment affected mostly oncogenic miRNAs while G2535 treatment affected both oncogenic miRNAs as well as tumor suppressor miRNAs, mostly members of the let-7 family, suggesting that these changes could reflect different mechanisms of action between these two anti-cancer agents. 

The Bioinformatics Core Facility at Wayne State University further analyzed this data in order to determine potential targets of the miRNAs that exhibited differential expression upon treatment. Due to the considerable number of potential targets for the miRNAs, we will limit our discussion by only focusing on miR-19b-1 of the miR-17-92 cluster as well as miR-221. Our analysis illustrated some potential targets that are consistent with the known targets as presented in Table **1**. For example, our analysis showed that miR-19b-1 may regulate the experimentally validated target Tsp1. It is also noted in Table **1** that the miR-17-92 cluster targets E2F1, a member of the E2F transcription factor family. We also found that miR-19b-1 may target E2F8, another related member of this transcription factor family which plays a role in inhibiting cellular proliferation. Thus miR-19b-1 may negatively regulate this transcription factor and allow proliferation to occur, contributing to the oncogenic role of the miR-17-92 cluster in cancer. Other potential miRNA targets found in our analysis provided support for the various functions of miRNAs as presented in Table **2**. This table identifies the miR-17-92 cluster which plays important roles in angiogenesis, apoptosis, proliferation, and tumorigenesis. Moreover, it is known that the various potential targets play significant roles in many of the same functions. For example, the targets Tsp1 and E2F8 mentioned above provide support for the angiogenic and proliferative functions of the miR-17-92 cluster in cancer, respectively. Additionally, it was reported that miR-19b-1 may also target caspase 8 (CASP8), death-associated protein kinase 3 (DAPK3), and programmed cell death 1 ligand 2 (PDCD1LG2), all genes known to be involved in apoptosis, further suggesting the anti-apoptotic function of the miR-17-92 cluster in cancer. 

The oncogenic miR-221 has been suggested to play an important role in apoptosis and proliferation and thus, various predicted targets were found to be involved in these processes. For example, our results showed that miR-221 may target BCL6 co-repressor (BCOR), BCL2-like 14 (apoptosis facilitator; BCL2L14), and programmed cell death 10 (PDCD10), all important genes that are known to regulate apoptosis. Negative regulation of these genes may impede their effects, suggesting the anti-apoptotic function of miR-221 in cancer. It was also found that miR-221 may target the WNT1 inducible signaling pathway protein 3 (WISP3) which is a tumor suppressor that has been shown to have inhibitory effects on cell proliferation and angiogenesis. Thus, negative regulation by miR-221 may counteract this tumor suppressor and allow these oncogenic processes to occur, supporting its suggested proliferative role in cancer. 

Overall, both B-DIM and G2535 are inhibitors of cancer cell growth and our preliminary results clearly suggest that these agents may execute their effects through pleiotropic mechanisms, especially involving the regulation of miRNAs. Decreased expression of oncogenic miRNAs upon treatment would most likely allow increased translation of their potential targets and cause induction of important cellular processes like apoptosis and inhibition of cell proliferation and angiogenesis. Conversely, increased expression of tumor suppressor miRNAs would most likely affect cellular processes in a similar manner, adding to the anti-cancer effects of these agents. Although these preliminary results are interesting, further investigations are needed to elucidate the roles of many of these miRNAs that could be mechanistically linked in mediating the effects of B-DIM and G2535 in cancer prevention and treatment in the future. 

## CONCLUSION

Since the discovery in 1993, miRNAs have been implicated as integral players in the biology of normal cells as well as diseased processes. Research over the last few years has allowed for the identification of various mechanisms and targets involving miRNAs, significantly improving our knowledge of cancer biology. Additionally, it has been determined that miRNAs may be extended to the clinic due to their potential value in diagnosis, prognosis, and therapy. Although this realm of research is very promising, it is important to remember that it does not come without obstacles. For example, off-target effects may potentially hinder the efficacy of therapy involving miRNAs [85]. Further research will most likely solve these types of problems and also identify more functional roles of miRNAs that could be exploited for cancer prevention and therapy. Finally, we believe that the future holds a great promise for the miRNA revolution in the fight against cancer and other health problems.

## Figures and Tables

**Table 1.** Various Oncogenic and Tumor Suppressor miRNAs

miRNA	Tumor Suppressor Activity	Oncogenic Activity	Expression in Cancer	Some Targets
Let-7 family	✓		-	RAS, PRDM1, HMGA2
miR-9		✓	+	PRDM1
miR-10a		✓	+	Tsp1
miR-15a/16-1	✓		-	BCL-2
miR-17-5p	✓		-	AIB1, E2F1
miR-17-92 cluster		✓	+	Tsp1, CTGF, E2F1, AIB1, TGFBR2
miR-21		✓	+	PTEN, TPM1, Pdcd4, maspin
miR-29b	✓		-	MCL-1, TCL-1
miR-34a	✓		-	E2F3
miR-106a		✓	+	RB-1
miR-124a	✓		-	CDK6
miR-127	✓		-	BCL-6
miR-141		✓	+	CLOCK
miR-142		✓	+	c-myc
miR-143	✓		-	Raf1, G-protein 7, ERK5
miR-145	✓		-	Raf1, G-protein 7
miR-146b		✓	+	KIT
miR-155/bic		✓	+	AT1R, TP53INP1
miR-181b	✓		-	TCL-1
miR-197		✓	+	ACVR1, TSPAN3
miR-200b		✓	+	PTPN12
miR-221		✓	+	KIT, p27(Kip1)
miR-222		✓	+	KIT, p27(Kip1)
miR-346		✓	+	EFEMP2
miR-372		✓	+	LATS2
miR-373		✓	+	LATS2

**Table 2.** Various miRNAs Grouped by Function

Function	miRNAs
Angiogenesis	*PRO:*miR-10a, miR-17-92 cluster, miR-155/bic
	*ANTI:*miR-34a
Apoptosis	*PRO:*miR-15a/16-1, miR-29b, miR-34a, miR-127
	*ANTI:*miR-17-92 cluster, miR-21, miR-155/bic, miR-221, miR-222
Invasion/Metastasis	*PRO:*miR-21, miR-197, miR-373
	*ANTI:*---
Proliferation	*PRO:*miR-17-92 cluster, miR-21, miR-141, miR-197, miR-221, miR-222, miR-346, miR-372, miR-373
	*ANTI:*let-7 family, miR-16-1, miR-17-5p, miR-34a, miR-143, miR-145
Tumorigenesis	*PRO:*miR-17-92 cluster, miR-372, miR-373
	*ANTI:*let-7 family

**Table 3.** Differential miRNA Expression when Comparing Panc-1 to HPDE*

Up-Regulated		Down Regulated	
Probe ID	Fold Diff.	Probe ID	Fold Diff.
hsa-miR-369-3p	7.45	hsa-miR-205	9.63
hsa-miR-376a	7.30	hsa-miR-200c	7.77
hsa-miR-196a	7.30	hsa-miR-200b	7.68
hsa-miR-493-5p	6.58	hsa-miR-224	7.04
hsa-miR-368	6.52	hsa-miR-148a	5.59
hsa-miR-495	5.93	hsa-miR-200a	5.57
hsa-miR-10b	5.55	hsa-miR-584	5.23
hsa-miR-10a	5.44	hsa-miR-575	5.05
hsa-miR-409-3p	5.38	hsa-miR-801	4.86
hsa-miR-432	5.28	hsa-miR-155	4.82
hsa-miR-379	5.12	hsa-miR-452	4.35
hsa-miR-487b	5.09	hsa-miR-141	4.30
hsa-miR-494	4.80		
hsa-miR-127	4.78		
hsa-miR-382	4.66		
hsa-miR-299-5p	4.44		
hsa-miR-329	4.42		
hsa-miR-493-3p	4.20		

* Table only includes the miRNAs with at least a four fold difference in expression.

**Table 4.** Differential miRNA Expression After Treatment in Panc-1*

Up-Regulated		Down Regulated	
Probe ID	Fold Diff.	Probe ID	Fold Diff.
**B-DIM TREATMENT**			
hsa-miR-663	1.97	hsa-miR-20b	0.82
hsa-miR-638	1.46	hsa-miR-17	0.58
hsa-miR-923	1.39	hsa-miR-106a	0.57
hsa-miR-565	0.92	hsa-miR-93	0.55
hsa-miR-30d	0.61	hsa-miR-221	0.55
		hsa-miR-20a	0.54
		hsa-miR-106b	0.45
		hsa-miR-222	0.42
**G2535 TREATMENT**			
hsa-miR-663	1.21	hsa-miR-34c-3p	2.37
hsa-miR-374b	0.96	hsa-miR-376a	1.04
hsa-miR-923	0.95	hsa-miR-196a	0.99
hsa-miR-638	0.94	hsa-miR-320	0.81
hsa-miR-30b	0.87	hsa-miR-99b	0.55
hsa-miR-365	0.74	hsa-miR-127-3p	0.51
hsa-miR-183	0.70	hsa-miR-382	0.49
hsa-miR-30d	0.59	hsa-miR-20b	0.48
hsa-miR-15b	0.54	hsa-miR-494	0.47
hsa-miR-30a	0.54	hsa-miR-106b	0.46
hsa-miR-425	0.53	hsa-miR-27b	0.46
hsa-miR-30c	0.48	hsa-miR-23a	0.45
hsa-let-7d	0.48	hsa-miR-93	0.41
hsa-let-7f	0.47		

* Table only includes miRNAs with at least a .4 fold difference in expression.

**Table 5.** Differential miRNA Expression After Treatment in Colo-357*

Up-Regulated		Down Regulated	
Probe ID	Fold Diff.	Probe ID	Fold Diff.
**B-DIM TREATMENT**			
hsa-miR-146a	0.71	hsa-miR-654-5p	1.67
hsa-miR-768-5p	0.62	hsa-miR-34c-3p	0.97
hsa-miR-27b	0.61	hsa-miR-19b	0.79
hsa-miR-26b	0.54	hsa-miR-663	0.65
hsa-miR-27a	0.47	hsa-miR-565	0.64
hsa-miR-125b	0.46	hsa-miR-638	0.60
hsa-miR-148a	0.43	hsa-miR-205	0.50
		hsa-miR-106a	0.40
**G2535 TREATMENT**			
hsa-miR-146a	1.68	hsa-miR-654-5p	2.94
hsa-miR-148a	1.39	hsa-miR-34c-3p	1.83
hsa-miR-374b	1.26	hsa-miR-663	1.67
hsa-miR-425	1.20	hsa-miR-19b	1.61
hsa-miR-191	0.99	hsa-miR-923	1.05
hsa-miR-768-5p	0.86	hsa-miR-20b	1.02
hsa-miR-182	0.84	hsa-miR-638	0.91
hsa-miR-128a	0.75	hsa-miR-106a	0.83
hsa-miR-361-5p	0.74	hsa-miR-221	0.81
hsa-miR-100	0.73	hsa-miR-565	0.72
hsa-miR-151-5p	0.64	hsa-miR-17	0.69
hsa-miR-15b	0.58	hsa-miR-149	0.68
hsa-miR-423-5p	0.58	hsa-miR-200b	0.55
hsa-miR-185	0.57	hsa-miR-205	0.54
hsa-let-7d	0.49	hsa-miR-98	0.49
hsa-miR-130b	0.47	hsa-miR-27b	0.47
hsa-miR-30d	0.47	hsa-miR-20a	0.44
hsa-miR-181a	0.46		
hsa-let-7b	0.44		
hsa-miR-200c	0.41		
hsa-let-7f	0.40		
hsa-miR-29a	0.40		

* Table only includes miRNAs with at least a .4 fold difference in expression.

## ABBREVIATIONS

miRNA: = MicroRNA
3’UTR: = 3’ Untranslated region
mRNA: = messengerRNA
pri-miRNA: = Primary miRNA
RNaseIII: = Ribonuclease III
pre-miRNA: = Precursor miRNA
TRBP: = *Trans-activator RNA (tar)*-binding protein
RISC: = RNA-induced silencing complex
CLL: = Chronic lymphocytic leukemia
Tsp1: = Thrombospondin-1
CTGF: = Connective tissue growth factor
TP53INP1: = Tumor protein 53-induced nuclear protein 1
PDAC: = Pancreatic ductal adenocarcinoma
PAM: = Prediction analysis of microarrays
Gli-1: = Glioma-associated antigen-1
